# Autologous hematopoietic stem cell transplantation in lymphoma patients is associated with a decrease in the double strand break repair capacity of peripheral blood lymphocytes

**DOI:** 10.1371/journal.pone.0171473

**Published:** 2017-02-16

**Authors:** Sandrine Lacoste, Smita Bhatia, Yanjun Chen, Ravi Bhatia, Timothy R. O’Connor

**Affiliations:** 1 Department of Cancer Biology, Beckman Research Institute, Duarte, California, United States of America; 2 Institute for Cancer Outcomes and Survivorship, School of Medicine, University of Alabama at Birmingham, Birmingham, Alabama, United States of America; 3 Division of Hematology and Oncology, School of Medicine, University of Alabama at Birmingham, Birmingham, Alabama, United States of America; University of California Davis, UNITED STATES

## Abstract

Patients who undergo autologous hematopoietic stem cell transplantation (aHCT) for treatment of a relapsed or refractory lymphoma are at risk of developing therapy related- myelodysplasia/acute myeloid leukemia (t-MDS/AML). Part of the risk likely resides in inherent interindividual differences in their DNA repair capacity (DRC), which is thought to influence the effect chemotherapeutic treatments have on the patient’s stem cells prior to aHCT. Measuring DRC involves identifying small differences in repair proficiency among individuals. Initially, we investigated the cell model in healthy individuals (primary lymphocytes and/or lymphoblastoid cell lines) that would be appropriate to measure genetically determined DRC using host-cell reactivation assays. We present evidence that interindividual differences in DRC double-strand break repair (by non-homologous end-joining [NHEJ] or single-strand annealing [SSA]) are better preserved in non-induced primary lymphocytes. In contrast, lymphocytes induced to proliferate are required to assay base excision (BER) or nucleotide excision repair (NER). We established that both NHEJ and SSA DRCs in lymphocytes of healthy individuals were inversely correlated with the age of the donor, indicating that DSB repair in lymphocytes is likely not a constant feature but rather something that decreases with age (~0.37% NHEJ DRC/year). To investigate the predictive value of pre-aHCT DRC on outcome in patients, we then applied the optimized assays to the analysis of primary lymphocytes from lymphoma patients and found that individuals who later developed t-MDS/AML (cases) were indistinguishable in their DRC from controls who never developed t-MDS/AML. However, when DRC was investigated shortly after aHCT in the same individuals (21.6 months later on average), aHCT patients (both cases and controls) showed a significant decrease in DSB repair measurements. The average decrease of 6.9% in NHEJ DRC observed among aHCT patients was much higher than the 0.65% predicted for such a short time frame, based on ageing results for healthy individuals.

## Introduction

Patients that undergo autologous hematopoietic stem cell transplant (aHCT) for the treatment of a persistent or relapsed/refractory Hodgkin lymphoma (HL) or non-Hodgkin lymphoma (NHL) are at high risk of a secondary therapy-related myelodysplasia/acute myeloid leukemia (t-MDS/AML), which constitutes a fatal complication of aHCT [1–7]. The major risk factors for t-MDS/AML (reviewed in [8] and [9]) include the cumulative dose of chemotherapeutic treatment to which individuals were exposed, especially alkylating agents and topoisomerase II inhibitors, as well as the use of high-dose total body irradiation as conditioning regimen for the aHCT [5,6,10–15].

Even among aHCT patients, the absolute risk of t-MDS/AML is still fairly low, with a measured incidence extending from 1.0% to 11.7% of patients (reviewed in [8]). Genetic factors could help explain why some individuals are more susceptible than others. In particular, differences related to DNA repair capacity (DRC) are expected to influence individual response and risk associated with exposure to chemotherapy during lymphoma treatment. Identifying patients at risk would be helpful in personalizing treatment course for each individual. Specific single-nucleotide polymorphisms have been linked to a higher risk of leukemogenesis after aHCT, most notably a specific polymorphism in *XRCC1*—a protein involved in multiple repair pathways related to its involvement in dealing with single strand breaks [16,17]—where carrying the allele A at rs25487 has been associated with a 4.5-fold increase in the risk of t-MDS/AML after aHCT [18]. Many other repair-related genes have also been connected to the risk of therapy-related secondary neoplasms [19–21]. However, for most individuals that develop t-MDS/AML, no genetic variant could be identified, showing that analyzing candidate genes involved in DNA repair has inherent limitations in identifying individuals at risk. Moreover, even when they could be identified, the functional significance of most genetic variants has not been determined in the previously published studies.

Another approach to identify individuals at risk is to measure functional DRC. DRC encompasses many cellular functions related to the maintenance of genome integrity. Both germinal and acquired changes could result in DRC variations, as they correspond to a functional assessment without assumptions regarding the underlying cause of the difference. It is generally understood that no single parameter can fully describe a person’s DRC and that analyzing DRC in multiple pathways is necessary to evaluate an individual’s repair capacity [22,23]. Pathway-specific DRC can be investigated by using host-cell reactivation assays where a template is damaged in a predetermined manner prior to introduction into the cells, at which point the repair is measured through the reactivation of the expression of a specific transgene. The pathway analyzed is determined by the design of the template and the type of damage incurred [24–31]. One general method used to validate a DRC assay is to verify that one can measure differences in repair when comparing cells of healthy individuals to cells of individuals with a known repair deficiency, for example XP cells (*i*.*e*., cells from individuals with xeroderma pigmentosum, which are deficient in nucleotide excision repair). Such comparisons have been used to validate DRC assays since the first investigation of interindividual differences in repair [24]. Hereditary repair deficiencies are indeed a form of interindividual difference in repair, but individuals carrying such DNA repair deficiencies show severe phenotypes compared to the general population, notably with increased risk of cancer at young age and accelerated aging [32,33]. Most individuals who will develop cancer in their lifetime do not carry such severe deficiency, but might still show differences in DRC. Therefore, identifying interindividual risk of malignancy is more likely to involve measuring subtle differences in repair proficiency than identifying actual repair deficiencies. This requires assays capable of distinguishing consistently subtle differences in repair.

To investigate DRC in individuals that underwent aHCT as a way to evaluate risk of developing t-MDS/AML, we used a systematic approach by first considering how to best measure interindividual differences in repair in healthy individuals. There is no absolute independent way to determine if any measurement accurately represent the DRC of a given individual, but assays that do measure interindividual differences in repair should consistently identify relative differences across cell types, if they are to represent the individual’s genetically-determined DRC rather than the specific cell type investigated. For aHCT patients in our cohort, we had access to cryopreserved peripheral blood cells, giving the possibility to analyze either directly primary lymphocytes (*i*.*e*., T cells induced or not to proliferate) or lymphoblastoid cell lines [LCLs] (*i*.*e*., B cells after EBV transformation). We have shown previously that we can obtain consistent measurements of repair when starting from identical frozen aliquots for both types of samples using identical host-cell reactivation assays [34]. LCLs constitute a quasi-infinite source of material and are a convenient model to investigate DRC for individuals, especially when several pathways are to be investigated [35]. There are however reports of lack of correlation in DRC measurements between LCLs and primary lymphocytes [36–38], leading to doubts regarding the capacity for transformed cells to predict DRC in primary cells.

Therefore, we first compared relative differences in repair in primary lymphocytes and LCLs derived from the same healthy individuals using host-cell reactivation assays for four specific DNA repair pathways: non-homologous end joining (NHEJ), single-strand annealing (SSA), base excision repair (BER) and nucleotide excision repair (NER). We confirmed that primary lymphocytes are likely a better model to analyze interindividual DRC and then applied the finalized assays to investigate DNA repair in primary lymphocytes of lymphoma patients that were known to have later developed or not t-MDS/AML. Patient samples analyzed were cryopreserved immediately before as well as at some time point after aHCT.

## Materials and methods

### Ethics statement

Use of human blood samples from healthy volunteers and patients undergoing aHCT was approved by the City of Hope Internal Review Board: IRB protocol #98117 entitled “The Molecular Pathogenesis of Therapy-Related Myelodysplasia/Acute Myelogenous Leukemia”. Patients and healthy volunteers provided their written consent.

### Patients’ samples

We have constructed a prospective cohort of patients undergoing aHCT for HL or NHL. Patients were followed longitudinally with collection of peripheral blood samples prior to aHCT, and serial peripheral blood samples until 5 years post-aHCT. This design allowed use of a nested case-control approach to compare DNA repair from “cases” that developed t-MDS/AML after aHCT with “controls” who did not develop t-MDS/AML after a period of follow-up that matched that of the corresponding index case. The matching criteria for control selection included underlying disease (HL or NHL), age at aHCT, race/ethnicity, and length of follow-up after aHCT (period of follow-up for the controls exceeded that of the patient cases under consideration). The selected individuals (Table 1) were analyzed for DRC before aHCT and at one time point afterwards, but prior to any t-MDS/AML diagnosis (S1 Fig). Repair data could not be obtained for every individual, time-point and/or every pathway, based on limited availability and/or the quality of the samples. The number of individuals included is indicated for each analysis. Five additional “control” patients were included in some analyses where cases/controls matching were not relevant (comparison pre *vs* post-aHCT for the same individual or comparison of patients to healthy individuals).

**Table 1:** Characteristics of aHCT lymphoma patients selected for DRC analysis.

	Cases		Controls	
**Primary diagnosis**				
HL (n, %)	2	14.3%	2	15.4%
NHL (n, %)	12	85.7%	11	84.6%
**Age at aHCT**				
(median, range)	57.5y (18-69y)		56y (21-68y)	
**Sex**				
Male (n, %)	10	71.4%	6	46.2%
Female (n, %)	4	28.6%	7	53.8%
**Race/ ethnicity**				
Non-Hispanic whites	11	78.6%	9	69.2%
Hispanics	3	21.4%	4	30.8%
Other	0	0.0%	0	0.0%

HL, Hodgkin Lymphoma

NHL, Non-Hodgkin Lymphoma

### Purification of Peripheral Blood Mononuclear Cells of healthy individuals (PBMCs)

To investigate DNA repair capacity in healthy individuals, blood samples (30-35ml in heparin tubes) were obtained from 16 healthy volunteers (numbered H33 to 48) and processed within 3h after being drawn. The blood was first diluted in 1 volume of Dulbecco's phosphate-buffered saline (DPBS) containing 2% heat-inactivated fetal bovine serum (FBS). The diluted blood was then transferred onto a SepMate-50 tube (Stem Cell Technologies) containing 15ml of HistoPAQUE-1077 (Sigma) or Lymphoprep (Stem Cell Technologies) and further processed as recommended by the tube manufacturer before freezing (see below).

### Hetastarch preparation of patients cells pre or post-aHCT

Peripheral blood of patients drawn before or after aHCT was prepared as to preserve all white blood cells (WBCs). The blood (30-35ml) was collected in heparin tubes and diluted in 1 volume of DPBS containing 2% heat-inactivated FBS. One volume of HESPAN Hetastarch (B Braun Medical Inc) was then added to the diluted blood to aggregate erythrocytes. After 45min of incubation, the upper phase containing WBCs was then transferred to a new tube and washed once with DPBS and once with culture medium before freezing (see below).

### Freezing and thawing of primary cells

For all primary cell preparations (PBMCs for healthy volunteers or all WBCs for patients), cells were frozen or thawed with the same protocol. Cells from ~2–3 ml of blood were resuspended in 1ml of Iscove’s Modified Dulbecco’s Medium (IMDM) containing 20% heat-inactivated FBS and 1ml of cold freezing medium (60% IMDM, 20% DMSO, 20% heat-inactivated FBS) was added. Temperature was progressively decreased to -80°C in a Mr Frosty container (Nalgene) and tubes were transferred the next day to the vapor phase of a liquid nitrogen storage tank.

For thawing, 30ml of IMDM (with 20% FBS) containing 4,000U/ml heparin and 62.5μg/ml DNase were added dropwise to a rapidly thawed aliquot and cells were allowed to recover for at least 2h in a 37°C incubator before a 15min spin at 200g and resuspension in RPMI 1640 medium containing 10% heat-inactivated FBS.

### Generation of lymphoblastoid cell lines

EBV transformation of 16 healthy individuals’ B cells into LCLs (S1 Fig) was performed as previously described [39]. Briefly, 1ml of EBV stock (supernatant prepared from B95-8 cells) was added to freshly thawed PBMCs (from 2-3ml of blood) resuspended in 2ml of culture medium (RPMI 1640 with 20% heat-inactivated FBS) containing 300μl of 100μg/ml PHA-P (Sigma). Cells were put in culture in a T25 flask and culture medium was refreshed twice a week, the volume being increased progressively based on cell density in the flask. LCLs used for repair experiments were frozen at a time point where they demonstrated their ability to grow after a freezing test (3–5 weeks post-infection in most cases). Freezing medium was 90% heat-inactivated FBS + 10% DMSO.

### Purification of T lymphocytes and induction of proliferation

When indicated, T lymphocytes were purified either from PBMCs or all WBCs (hetastarch) samples using Dynabeads Flowcomp human CD3 kit (Life Technologies) as recommended by the manufacturer with the following modifications (the protocol was scaled up proportionally when there were more cells in the sample). Up to 5 x 10^6^ total cells were resuspended in 50μl of cold isolation buffer (DPBS with 2% heat-inactivated FBS and 2mM EDTA) in a 2ml tube and 2.5μl of FlowComp Human CD3 antibody were added and incubated with the cells 10min at 4°C. Excess antibody was then washed out with 500μl of isolation buffer and cells were pelleted by centrifugation at 350g for 8min at room temperature. The cell pellet was then resuspended in 130μl of isolation buffer and 10μl of prewashed FlowComp beads were added and kept in suspension in the solution for 15min at room temperature by gentle agitation using a Hula Mixer (Life Technologies). Cells bound to the beads were then recovered by adding of 130μl of isolation buffer mixed well with 2–3 up and down pipet motions and then placing the tube on a DynaMag-5 magnet (Life Technology) for 2min. While still on the magnet, all buffer (containing unbound cells) was then removed from the tube and bead-bound cells were then washed a second time with 130μl of isolation buffer before being resuspended in 150μl of the release buffer provided with the purification kit (incubation 10min under gentle agitation with Hula Mixer at room temperature). T cells released from the beads were then counted using Trypan blue and washed by adding 500μl of isolation buffer and centrifugation 350g for 8min. Cells were then resuspended in an appropriate volume of culture medium (RPMI 1640 + 10% heat-inactivated FBS). When indicated, T cells were then induced to proliferate by adding 1:1 ratio of CD3/CD28 DynaBeads human T activator (Life Technologies) and 30U/ml of human recombinant IL-2 (PreproTech) as recommended by the manufacturer. Growth culture volume for the induction of proliferation was 500μl (24-well plate) for all healthy donor samples (1.7–8.0 x 10^5^ T cells total) or 100μl (96-well plate) for 5 x 10^4^ T cells for patient samples.

### Host-cell reactivation assays for double-strand break repair capacity

**Fig 1:**
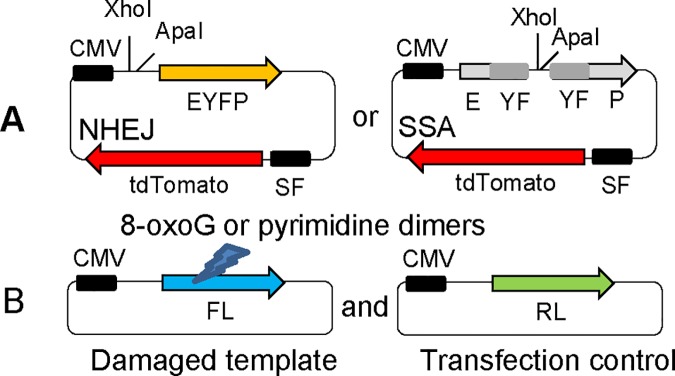
Plasmids used for host cell reactivation assays. **(A)** pSF-tdTomato-END (for NHEJ, left) and pSF-tdTomato-HOM (for SSA, right) plasmids used for DSB repair assays. XhoI+ApaI double-digestion (= DSB) generated *in vitro* is repaired by either NHEJ or SSA after transfection in cells, thereby restoring EYFP expression. The common sequence between the two overlapping halves of EYFP in the SSA template (“YF” in grey) is 350bp long. Repair is measured by the percentage of EYFP^+^ cells among tdTomato^+^ cells. **(B)** pM1-Luc plasmid (left) and pRL-CMV (right) plasmids. Oxidative (8-oxoG) or UV damage (pyrimidine dimers) generated *in vitro* on pM1-Luc plasmid impairs expression of firefly luciferase (FL) that is then restored by BER or NER, respectively, once transfected in cells. Repair is measured by the FL activity after normalization to renilla luciferase (RL) activity (transfection control) and in comparison to the undamaged FL template (100% expression) in the same cells.

Plasmids pSF-tdTomato-END for NHEJ repair and pSF-tdTomato-HOM for SSA repair (Fig 1A) were digested with XhoI and ApaI and complete double-digestion was verified for quality control as described previously [34]. For all cell types investigated, DSB repair assays were performed using 400ng of either END (for NHEJ) or HOM (for SSA) linearized plasmids transfected into cells using the P3 Primary cells nucleofection kit on the 4D Nucleofector system (Lonza). Electroporations were performed using the 20μl format (strips of electrocuvettes) and the EO-115 program. Cells were then recovered in 180μl of RMPI 1640 with 10% FBS in a 96-well plate and placed in an incubator for 12h. To determine repair efficiency, cells were then resuspended in HBSS containing 0.3μg/ml DAPI and the proportion of EYFP^+^ cells among viable (DAPI negative) transfected cells (tdTomato^+^) determined using a BD LSRFortessa cell analyzer with the FACSDiva software (version 6, BD Biosciences) as described previously [34]. NHEJ and SSA repair were analyzed in parallel and in triplicates for each sample. The number of technical replicates (separate transfections of the same cells with the same construct in the same experiment) was in some cases reduced to 1 or 2 for patient samples, based on the number of lymphocytes available.

### Host-cell reactivation assays for BER or NER capacity

BER and NER assays use the same plasmid as reporter (Fig 1B) and differ only by the nature of the damage applied to the firefly (*Photinus pyralis*) luciferase (FL) reporter gene that is reactivated upon repair (in the pM1-Luc plasmid). The pRL-CMV plasmid expressing the renilla (*Renilla reniformis*) luciferase is used as an internal control to normalize FL activity levels for transfection efficiency (see below for description of the assay).

To prepare the BER test plasmid, 250μg of pM1-Luc in a final volume of 2.5ml in 10mM NaPO_4_ pH 7.5 buffer was placed in a small weighing dish on ice and irradiated for 5min with a pre-warmed 150W incandescent bulb at a distance of 10.5cm in presence of 15μM methylene blue, which generated 8-oxoG damage. For the NER test plasmid, 250μg pM1-Luc in a final volume of 2.5ml in 10mM NaPO_4_ pH 7.5 buffer was placed in a small weighing dish on ice and irradiated with 120J/m^2^ with a germicidal (UVC) lamp to generate pyrimidine dimers. The irradiation time necessary to provide the UVC dose was determined based on the radiant incidence of the lamp measured that day using a UVX digital radiometer (UVP).

After exposure to DNA damage, both BER and NER plasmids were precipitated with 1/10^th^ volume of 3M NaOAc pH 5.2 and 2.5 volumes of ice cold 100% ethanol. After a wash with 70% ethanol, DNA pellets were resuspended in 500μl H_2_O and the DNA concentrations verified with a Nanodrop spectrophotometer. Finally, the volume of H_2_O was adjusted to a final concentration of 400ng/μl. To verify the level of DNA damage present in BER and NER plasmids, we designed a method to estimate the level of inhibition of polymerase extension specifically in the coding sequence of the FL reporter gene (S2 Fig). BamHI-digested templates (site in 3’ of the end of the gene) were subjected to 5 cycles of primer extension starting from a Cy5.5 labeled CMV-F primer. The level of Cy5.5 signal at full length extension indicates the proportion of undamaged template that remains and can serve as quality control for each batch of plasmid preparation. The BER and NER plasmid batches used for this study showed 16% and 27% of residual full length extension, respectively (S2 Fig). Although this does not predict directly the level of gene expression *in vivo*, the damage frequency present in these plasmids is expected to result in some background expression of the undamaged FL gene once in cells, even if no repair were to occur. Beyond the opportunity for a quality control for the level of damage generated, the advantage of such a level of damage is that we can infer from these estimates that few lesions exist (1 or 2) per template. As a consequence, a limited number of discrete repair events can be expected to result in increased expression levels, making the assays sensitive to low levels of repair.

To measure DRC, 400ng of either BER or NER template were co-transfected (see nucleofection protocol in previous section) with 100ng of pRL-CMV plasmid. Pre-mixes containing this ratio of BER+pRL-CMV or NER+pRL-CMV plasmids were prepared in advance to insure consistency in co-transfected amounts, eliminating pipetting error as a source of variation between transfections. After nucleofection, cells were transferred into 180μl phenol red-free RPMI 1640 medium with 10% heat-inactivated FBS and placed in an incubator for 8h. To quantify repair by BER or NER, cells were centrifuged in the 96-well culture plate and some of the excess culture volume was removed. Finally, 75μl of resuspended cells were used to measure the firefly over renilla luciferase relative activities for each transfection, using the Dual Glo Luciferase assay system (Promega) as recommended by the manufacturer. pRL-CMV plasmid alone and undamaged pM1-Luc+pRL-CMV were used as controls for 0% and 100% firefly luciferase activity, respectively. Two (patient samples) or 3 (healthy controls) technical replicates were averaged to determine DRC, meaning separate test transfections of the same construct in the same experiment. The reproducibility among those replicates tended to be lesser in samples where more cell death was observed (analysis in patient samples and/or at time points more than 8h post-transfection).

### Statistical methods

Data obtained for all the assays are summarized (S1 File.). Pearson’s correlation was used to assess the associations between DRC in primary T cells and LCLs. Nonparametric Wilcoxon signed rank test was used to compare DNA repair between matched pairs of cancer case and cancer control before and after transplant. Nonparametric Mann-Whitney-Wilcoxon test was used to compare difference in DNA repair between cancer group and healthy controls. Multivariate linear regression was used to assess the difference between transplant patients at pre-HCT and healthy controls, adjusting for age. Detailed methods are mentioned with the corresponding results in the text, tables or figure legends. Bonferroni correction was used to adjust multiple comparisons of the different outcomes.

PROC CORR, NPAR1WAY, GLM of SAS software, version 9.4 (SAS Institute, Cary, NC) or GrapPad Prism 6 were used for analysis. Two-sided tests with p<0.0125 were considered statistically significant with Bonferroni correction for multiple comparisons.

## Results

### Non-induced lymphocytes are a good model to investigate interindividual differences in DSB repair capacity

To determine if transformed cells (LCLs) are a good proxy for DSB repair in primary lymphocytes of the same healthy individual, repair was evaluated using host-cell reactivation assays after transfection of damaged plasmids into both cell types using the same basic protocol. DSB repair capacity by NHEJ or SSA was measured by the proportion of EYFP^+^ cells among transfected cells (tdTomato^+^), as determined by flow cytometry. EYFP expression is the result of NHEJ repair or SSA repair, depending on the design of the linearized plasmid transfected (Fig 1A).

We have shown before that NHEJ and SSA repair can be measured in non-induced lymphocytes, even when mixed with other white blood cells, as the latter can be disregarded from the flow cytometry analysis [34]. However, many protocols investigating repair in peripheral blood lymphocytes induce cell proliferation prior to DNA repair analysis and we first wanted to determine whether or not to induce lymphocytes prior measuring NHEJ and SSA repair. We opted to induce T lymphocytes using CD3/CD28 beads (Dynabeads human T activator CD3/CD28, Invitrogen) that mimic physiological activation of T cells when recognizing a specific antigen. Such an induction is better controlled when performed on purified T (CD3^+^) cells with a ratio of one bead per T cell. Experiment on the effect of induction were therefore performed on purified T cells rather than PBMCs as a whole, but we have verified that DSB repair is constantly the same for a given individual whether lymphocytes were purified or not prior to repair measurements (S3 Fig). This result also confirms that repair measured directly in PBMCs with this protocol is a good representation of repair specifically in T cells and therefore that T cells are the cell type investigated in all our experiments performed on primary lymphocytes regardless of the type of sample preparation.

**Fig 2:**
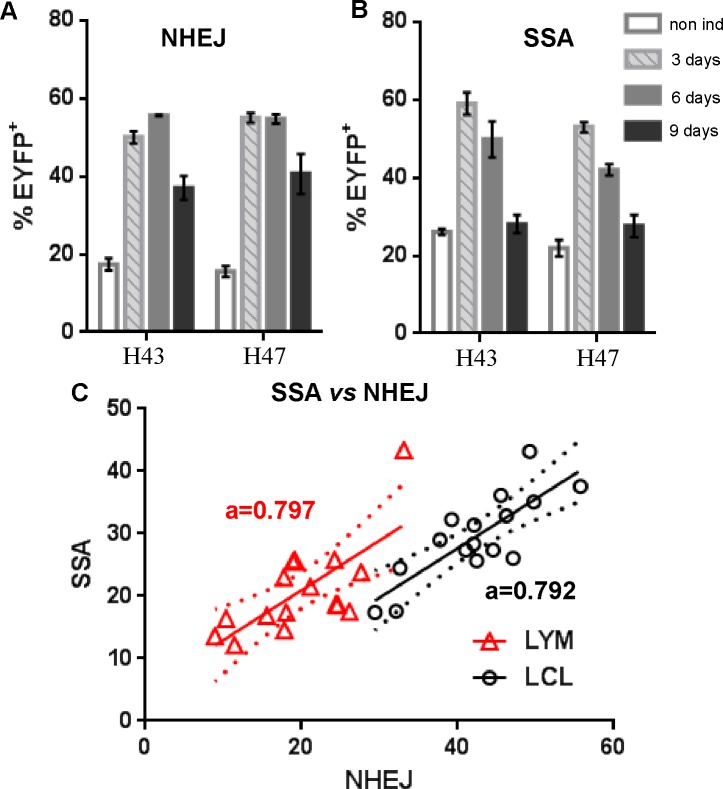
DSB repair capacity in lymphocytes and LCLs of healthy individuals. **(A)** NHEJ and **(B)** SSA repair in purified T cells of 2 healthy individuals either non-induced or induced to proliferate for 3 to 9 days **(C)** Correlation between SSA and NHEJ repair in non-induced unpurified lymphocytes (red triangles) and in 3–5 weeks old early LCLs (black circles). The linear regression trend lines with 95% confidence intervals are indicated, as well as the value of the slopes (a).

To determine the effect of induction on DSB repair, purified T (CD3^+^) cells of two healthy individuals were either analyzed non-induced or induced to proliferate for up to 9 days. Fig 2 shows a strong increase in both types of DSB repair upon induction of proliferation (Fig 2A and 2B for NHEJ and SSA, respectively), but the levels of induction greatly varied with the time-point analyzed. Such variations make it difficult to conclude which, if any, of the obtained values might best represent the individual’s DRC, as the time-point selected post-induction would tremendously influence the results for any given individual. Moreover, the effect of the induction is so strong (up to 3.5 times the non-induced value after 3 days induction) that it is likely to dwarf the effect of any existing interindividual differences in repair. As a result, we opted to use non-induced cells for DSB repair assays on primary lymphocytes of healthy individuals.

Repair experiments in LCLs were performed on freshly thawed, early LCLs (frozen shortly after confirmation of their transformed status ~3–5 weeks post-EBV infection). Table 2 shows that DSB repair measurements in non-induced lymphocytes of 16 individuals as compared to LCLs derived from the same individuals are positively correlated, but that correlation does not reach significance. As uninduced primary lymphocytes most closely represent cells directly from the individual investigated, they are the most likely to represent the individuals’ DRC when compared to LCLs.

**Table 2:** Correlation between primary lymphocytes and LCLs for each repair pathway.

Pathway	n	r	p-value
NHEJ	16	0.397	0.128
SSA	16	0.368	0.161
BER	12	0.158	0.624
NER	12	0.269	0.398

r, Pearson’s correlation coefficient

In the course of measuring DSB repair in non-induced lymphocytes and LCLs, we noticed that the two DSB repair pathways seemed to vary together for each cell type. Table 3 shows that there is a significant correlation between NHEJ and SSA measurements (r = 0.708 and r = 0.807 for lymphocytes and LCLs, respectively) and Fig 2C illustrates the direct relationship between the two types of repair in both cell types. The identical slopes for the trend lines of lymphocytes and LCLs indicate that there is a pattern in the relationship between those pathways that exists systematically in cells, indicating that measurements in LCLs might be meaningful, even if the LCLs do not fully recapitulate interindividual differences in repair.

**Table 3:** Correlation between DRC by NHEJ and SSA and between BER and NER for all types of samples investigated.

	NHEJ *vs* SSA		NER *vs* BER	
	r	p	r	p
Healthy lymphocytes	0.708	0.002	0.918	9.91E-06
Healthy LCLs	0.807	0.0002	0.819	0.001
Pre-aHCT patients	0.345	0.161	0.598	0.007
Post-aHCT patients	0.676	0.0003	0.478	0.012

r, Pearson’s (healthy samples) or Spearman’s (patient samples) correlation coefficient

### Induction of T cells for proliferation is necessary to investigate interindividual differences in BER and NER repair capacity

BER and NER repair activity were measured using the capacity to reactivate the firefly luciferase reporter expression that is inhibited by 8-oxoG or pyrimidine dimers damage generated *in vitro* prior to transfection (Fig 1B).

**Fig 3:**
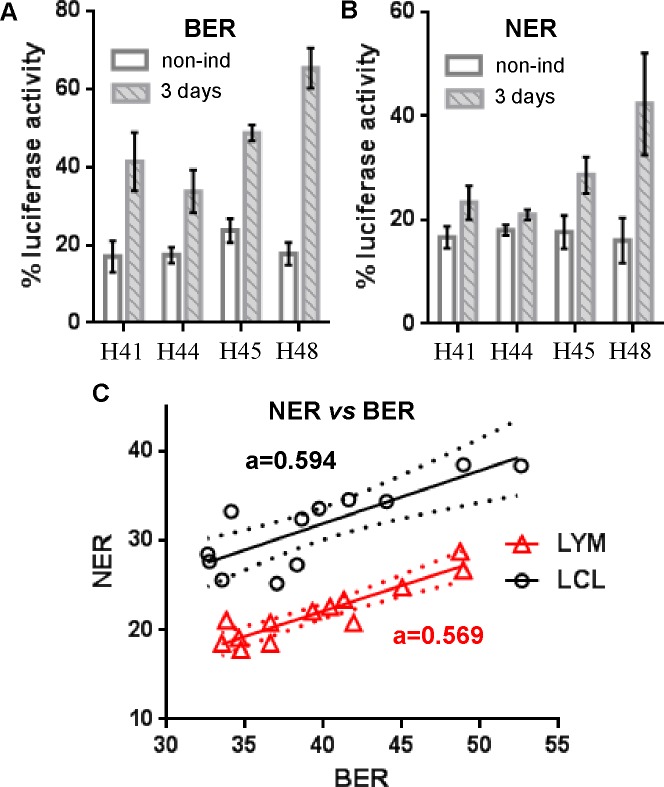
BER and NER repair capacity in lymphocytes and LCLs of healthy individuals. **(A)** BER and **(B)** NER repair in purified T cells of 4 healthy individuals either non-induced or induced to proliferate for 3 days. Luciferase expression in non-induced samples is consistent with background expression from undamaged template (S2 Fig) **(C)** Correlation between BER and NER repair in T cells induced 3 days (red triangles) and in early LCLs (black circles) for 12 individuals. The linear regression trend lines with 95% confidence intervals are indicated, as well as the value of the slope (a).

As for the previous assays, we first verified the effect of CD3/CD28-induced proliferation on BER and NER activity in purified T (CD3^+^) cells (Fig 3A and 3B), but found that only background levels of luciferase activity (expression due to undamaged template that is similar for all individuals–S2 Fig) could be detected without induction, and therefore that induction was necessary to investigate those repair pathways. We opted to measure BER and NER after activation performed in a controlled manner using an exact 1:1 ratio of T cells to CD3/CD28 beads, and found that we could reliably detect interindividual differences in BER and NER using these experimental conditions (3 days induced samples in Fig 3A and 3B).

Therefore we compared BER and NER in the same early LCLs as previously, and in T cells induced to proliferate for 3 days and found there was no correlation between the two cell types for those pathways either (Table 2). On the other hand, and similar to what was observed for DSB repair assays, Table 3 shows that there is a significant correlation between BER and NER measurements (r = 0.918 and r = 0.819 for lymphocytes and LCLs, respectively) and Fig 3C illustrates the direct relationship between the two types of repair measurements in each cell types. The slopes for the trend lines are similar as well, indicating that those pathways follow also a pattern in their relationship to each other in all cells analyzed.

Overall, and although there is evidence that an individual’s genetic make-up contributes to the measured repair and that there are clear patterns to repair as determined in LCLs, it seems likely that primary cells allow more accurate measurements of interindividual differences in DNA repair capacity.

### DNA repair capacity in purified T cells of aHCT patients

Our main purpose in this study was to analyze potential differences in repair in individuals that underwent aHCT, but the frozen samples we have available for patients in this cohort contain all white blood cells, not just PBMCs, as they were simply purified from red blood cells using hetastarch rather than a density gradient. We have shown previously that repair in lymphocytes can be influenced by the presence of granulocytes in their environment at the time of transfection, as is the case in hetastarch-prepared samples [34]. Moreover, we had very limited amount of material for each lymphoma patient. Therefore, all repair analysis on patient samples were performed on purified T cells (CD3^+^). After purification, a limited number of cells (5 x 10^4^ T cells) were expanded using CD3/CD28 beads and IL-2 for 3 days for BER and NER assays, whereas the rest of the purified T cells were used non-induced for DSB repair assays (work flow scheme in S4 Fig). This strategy allowed repair measurement for all 4 DNA repair pathways using a single frozen aliquot representing cells from 2–3 ml of blood.

Patients in the cohort were recruited prior to aHCT and followed for up to 5 years afterwards. Therefore, we had multiple samples available for each individual, taken at different time points during the study. We decided to analyze 1) cells drawn prior to the transplant in order to determine if DRC could help in any way predict which individuals would later develop t-MDS/AML and 2) cells drawn for the same individuals at one time point post-aHCT but prior to any malignancy diagnosis, for DRC after transplantation (S1 Fig). Cases (who later developed t-MDS/AML) were matched to paired control individuals from the same cohort based on their diagnosis, ethnicity and duration of follow up for the post-aHCT sample (Table 1). Table 4 shows the paired comparisons between cases and controls regarding their DRC for all 4 pathways. No type of repair was significantly different between cases and controls, indicating that there was no measurable deficiency in DRC specifically in aHCT patients that later developed t-MDS/AML.

**Table 4:** Comparison of DRC between cases (t-MDS/AML) and controls for all pathways pre or post-aHCT.

	Cases		Controls		
	n	median (range)	n	median (range)	p-value
**Pre-aHCT**					
NHEJ	8	22.8 (14.4–49.4)	8	23.5 (21.4–33.5)	0.196
SSA	9	18.3 (12.3–51.5)	9	16.6 (12.6–26.4)	0.570
BER	9	33.2 (6.6–88.5)	9	24.0 (11.0–51.9)	0.164
NER	9	27.8 (12.1–62.8)	9	22.7 (12.3–47.3)	0.910
**Post-aHCT**					
NHEJ	10	14.6 (10.7–33.0)	10	15.9 (10.5–29.9)	0.846
SSA	10	14.7 (9.8–22.1)	10	11.9 (8.5–22.0)	0.625
BER	13	32.2 (14.1–76.3)	13	32.5 (11.1–54.6)	0.735
NER	13	20.3 (13.5–51.3)	13	22.5 (10.2–47.5)	0.542

DRC values are in %.

p-values are for Wilcoxon signed rank test

### Transplantation is associated with a decrease in DSB repair capacity

**Fig 4:**
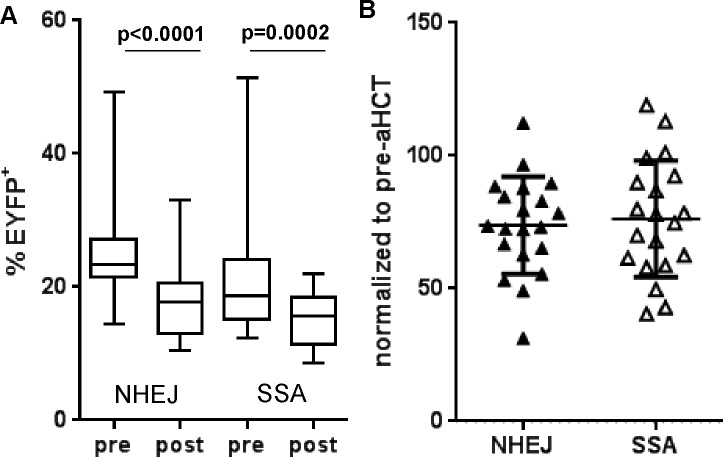
DSB repair decreases after aHCT. **(A)** NHEJ and SSA measured in the same 20 aHCT recipients (13 controls, 7 cases) before and after aHCT (p-value for Wilcoxon signed rank test). **(B)** Repair post-aHCT normalized to pre-aHCT for each patient. A value <100% indicates a decrease in NHEJ (close triangles) or SSA (open triangle) after transplant.

For 20 aHCT recipients (13 controls and 7 cases), we could obtain DSB repair data both before and after aHCT (S1 Fig), allowing us to study whether the transplant itself had affected their DRC. We observed a significant decrease in both NHEJ and SSA after aHCT (Fig 4A) with an average decrease of 6.9% and 6.1% in repair, respectively. Most individuals showed a decrease in DSB repair (relative repair <100%) with an average post-aHCT repair equivalent to 73.8% and 76.2% of its value pre-aHCT for NHEJ and SSA, respectively (Fig 4B). Cases did not show more decrease than control individuals (data not shown) and the observed DSB repair decrease did not help predict what individuals were at risk of developing t-MDS/AML.

In contrast to the results with NHEJ and SSA, BER and NER repair analyzed for 18 individuals (9 cases and 9 controls) was not significantly affected by aHCT. The results for NER and BER do not indicate that repair for individuals were actually identical before and after transplant, but rather that observed variations did not display a specific identifiable pattern (S5 Fig).

### Influence of age on repair capacity

**Fig 5:**
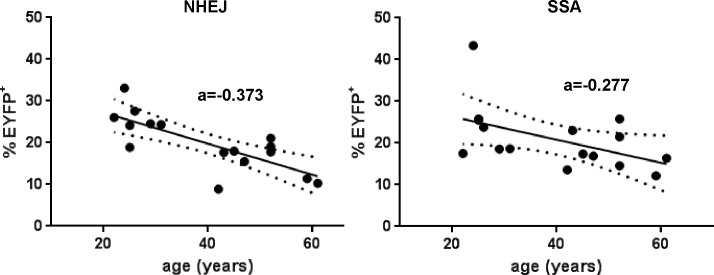
DSB repair capacity decreases with age in non-induced lymphocytes of healthy individuals. Trendline and 95% confidence interval are shown for NHEJ (left) and for SSA (right), as well as the slope of the trendline (a)

We wondered if a decrease in repair could be a manifestation of aging in immune system cells. To investigate if repair was affected by age, we analyzed again the repair results for healthy individuals for each type of repair pathway in function of age at blood draw (Table 5) and found that both types of DSB repair capacity are inversely correlated with age, and more specifically so for NHEJ repair (Fig 5). Based on the slope (a) of the trendline, NHEJ repair can be estimated to decrease at a rate of 0.37% per year in healthy individuals.

**Table 5:** Correlation between repair and age at blood draw in healthy individuals and pre-aHCT patients.

	Healthy individuals		Pre-aHCT patients	
	r	p-value	r	p-value
NHEJ	**-0.764**	0.0006	0.004	0.985
SSA	-0.503	0.047	-0.253	0.186
BER	-0.030	0.922	-0.135	0.502
NER	0.116	0.706	0.069	0.732

r, Pearson’s correlation coefficient

Correlation with significant p-value indicated in bold

On the other hand, neither BER nor NER were associated with age in healthy individuals (S6 Fig). Overall, the data is consistent with DNA repair capacity in NHEJ and, to a lesser extent in SSA, being a function that decreases with age in non-induced lymphocytes. Interestingly, this relationship of DSB repair to age was not statistically significant when analyzing LCLs of the same individuals (data not shown), with a correlation of -0.415 for NHEJ (p = 0.110) and -0.094 for SSA (p = 0.730).

### Comparison of repair in patients *vs* healthy individuals

**Fig 6:**
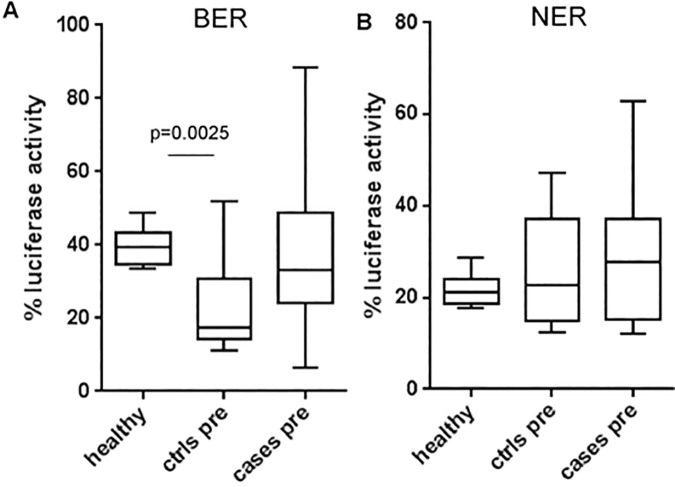
DRC in healthy individuals compared to pre-aHCT patients who later developed t-MDS/AML (cases) or not (ctrls). (**A**) BER and (**B**) NER. p-value is for Mann-Whitney test.

We wondered how pre-aHCT patients compared to our group of healthy individuals regarding their DRC. BER was lower in pre-aHCT patients as compared to healthy individuals (p = 0.0134) but NER was not (p = 0.4347). Especially the subgroup of control patients, those who never developed t-MDS/AML, showed a much lower BER capacity than healthy individuals (average 23.3% and 39.5%, respectively–p = 0.0013) (Fig 6). After adjustment for age at aHCT, NHEJ repair in patients was marginally higher than in healthy individuals (p = 0.0149), but SSA repair was not (p = 0.4287). Interestingly, although patient samples post-aHCT followed the same patterns identified for healthy individuals (Table 3), with a correlation between NHEJ and SSA (r = 0.676, p = 0.0003) on the one hand and between BER and NER on the other hand (r = 0.478, p = 0.012), pre-aHCT patients did not show the correlation between NHEJ and SSA, which was mostly lost (p = 0.345, p = 0.161). Moreover, and unlike in healthy individuals, neither NHEJ nor SSA in pre-aHCT patients displayed any relationship to the person’s age at time of aHCT (r = 0.004, p = 0.985 and r = -0.253, p = 0.186, respectively–Table 5). Overall, these results indicate that pre-aHCT DRC measurements have features that are not consistent with those observed for other samples, including repair for the same individuals after transplantation.

## Discussion

It is likely that differences in DRC influence an individual’s risk of t-MDS/AML after aHCT. Being able to measure DRC might therefore help in predicting who is at higher risk and how to adapt their treatment accordingly. DRC in peripheral blood cells can be investigated directly in primary (mostly T) lymphocytes, or in LCLs after EBV transformation. Before deciding which cell model to use for measuring DRC in aHCT patients, we first investigated what seemed the most reliable way to determine an individual’s DRC using samples obtained from healthy individuals.

Unsurprisingly, the measurement of interindividual differences in BER and NER repair in primary lymphocytes was found to be possible only in lymphocytes that were induced to proliferate. This requirement was observed the first time a host cell reactivation assay was performed on primary lymphocytes to measure NER [24]. However, the fact that background expression levels of luciferase expression can still be measured in non-induced cells confirms that this requirement is due to a true lack of significant repair in non-induced cells, and not just an issue with transfection or measuring low level of reporter activity.

There is, however, no absolute requirement to induce proliferation in order to measure DSB in primary lymphocytes [40], at least for the NHEJ and SSA pathways that we found to be functional in non-cycling cells [34]. Still, many DSB repair assays in lymphocytes published in the literature have been performed in induced cells [30]. In many cases, it is because one of the objectives is to measure homologous recombination (HR) that will only occur in cycling cells during the S phase [41,42]. However, the choice to induce cells for proliferation rather than use them uninduced might sometimes also have been based on the assumption that non cycling cells are generally not proficient at DNA repair [43,44]. Our own data showed that DSB repair is much more efficient (~ 3 fold) in induced cells compared to non-induced ones (Fig 2A and 2B), but the DRC varied with time post-induction, making it unclear what factor(s) might be controlling the level of repair in induced cells. Analyzing SSA repair (a sub-pathway of HR) in non-cycling cells might not be a perfect proxy of the DRC in HR for the individual, but comparing NHEJ and SSA of an identical DSB (with ApaI and XhoI overhangs in both cases) should be able to address the question of whether cells show preference in resolving the DSB towards end-joining (NHEJ) *vs* a homology-directed pathway that first requires strand resection (SSA). Those two systems are thought to be in competition with each other for the repair of the same breaks, the choice between direct end-joining and strand resection being thought to be predictive of preferential repairs in an error-prone or error-free manner, respectively (recent reviews in [45–48]). Although SSA repair is obviously error-prone as it leads to deletions, our results contradict this notion that end-joining and strand resection are in direct competition for the same DSB repair in the cells we analyzed. Low NHEJ is never associated with a relatively high SSA and in fact, NHEJ and SSA are clearly positively correlated to each other in both primary lymphocytes and LCLs (Fig 2C). A recently identified sub-pathway of NHEJ (alt-NHEJ) has been shown to use strand resection and micro-homologies of a few nucleotides for the repair of DSB breaks by end-joining [49]. This constitutes a possible functional connection between SSA and end-joining repair if alt-NHEJ were to be the main mechanism of NHEJ in our experimental system. Analyzing multiple repair events at the sequence level should help determine if micro-homologies were used and therefore if alt-NHEJ is a major contributor to the observed repair.

When we compared primary lymphocytes to the LCLs obtained from the same individual, no significant correlation in either NHEJ or SSA repair could be found (Table 2). Although repair in LCLs might still represent the individual’s DRC to a certain extent, this is consistent with some previous work that indicated that LCLs might not reflect accurately repair in primary cells [36–38].

An intriguing result from DSB repair measurements in non-induced lymphocytes of healthy individuals was that both NHEJ and SSA repair were inversely correlated to the age at time of blood draw of the individual (age range 22–61 years–Fig 5). This result is consistent with a previous demonstration that NHEJ decreased in aging mice using a transgenic model [50] and the observation of a trend towards a decline in DSB repair with age in human primary lymphocytes as measured with the neutral Comet Assay and γH2AX response after γ–irradiation [51]. However, the latter results required the analysis of more than 200 individuals (in non-induced peripheral blood cells) and some sophisticated statistical analysis to ascertain the relation of repair to age. In our case, the raw data (the % EYFP positive cells) for NHEJ repair in cells of 16 individuals was sufficient to see an inverse correlation with age of the donor r = -0.764 (p<0.0006), indicating that host cell reactivation is likely a better method to investigate the effect of age on NHEJ repair. Interestingly, NHEJ was globally much higher in LCLs (~2 fold, see shift of the trendline towards higher NHEJ in Fig 2C) than in non-induced lymphocytes and the correlation of repair to age was mostly lost in LCLs derived from the same individuals. This indicates that changes of NHEJ with age might not be irreversible, but possibly regulatory in nature. Overall, our data suggest that NHEJ and SSA are closely co-regulated together, and that the level of repair can be affected by age, the induction of proliferation and/or the EBV transformation. The mechanism of DSB repair regulation in cells remained to be determined but always showed a certain consistent pattern when investigating the same cell type, as manifested by the identical slopes observed in the relationship of NHEJ to SSA (Fig 2C).

We found a similar close relationship between DRC by BER and NER with a strong correlation found in both primary induced T cells and in LCLs (Table 3 and Fig 3C), but once again with a lack of correlation when comparing repair in primary lymphocytes and LCLs of the same individuals (Table 2). BER and NER assays are very similar in nature as they both measure the inhibition of luciferase transcription on the very same template caused by different types of transcription-blocking DNA modifications (8-oxoG for BER, pyrimidine dimers for NER). They were also systematically measured using separate parallel transfections in the same experiment and we cannot exclude that the observed correlation is the result of a technical bias related to the technique of host-cell reactivation itself (*e*.*g*., measuring a function related to the capacity of expressing transgene from a plasmid template after transfection rather than differences in repair *per se*). This explanation is unlikely, though, as the level of background luciferase expression was similar for all individuals in non-induced T cells for both BER and NER templates (Fig 3A and 3B). As a result, the ability to measure luciferase activity in lymphocytes seemed comparable between individuals and only the induction of repair (by cell activation) allowed the detection of differences in luciferase activity between individuals. The BER to NER correlation could also be the effect of a cellular function other than repair that affects both assays in the same manner, and that could then be measured by two separate methods. For example, an individual’s capacity to perform transcriptional bypass on damaged template could have such an effect on both BER and NER assays, and RNA polymerase II does transcribe through 8-oxoG [52], and even through CPDs [53,54]. However, the fact that the correlation was also there in LCLs (Fig 4C), even if there was no correlation between primary lymphocytes and LCLs for the same individuals (Table 1) suggests that BER and NER, just like NHEJ and SSA, are simply co-regulated in a manner that is not entirely understood, so that when one type of activity is enhanced, so is the other. As a result, interindividual differences that we measured might reflect interindividual differences in the regulations of the pathways rather than in repair efficiency itself.

A decrease in NER with age has been shown previously in human fibroblasts, which was associated with a decrease in DNA repair-related gene expression [55,56]. We did not find any association of BER nor NER with age in our samples (S6 Fig), but the age-related differences might be have been masked in lymphocytes by the need to induce the cells for proliferation to analyze for those types of repair.

Finally, we analyzed the DRC for all 4 pathways in patients’ T lymphocytes (non-induced for NHEJ and SSA, induced for BER and NER) either before or after aHCT. Identifying individuals that are most at risk before the transplant would be most relevant as the treatment could be adapted accordingly. We did not find any form of DRC that was significantly different in individuals that later developed t-MDS/AML (cases), as compared to matched individuals that did not (controls). There were some differences, though, that could be identified in pre-aHCT patients when compared to the group of healthy individuals. NHEJ was marginally higher in patients (cases and controls combined) than in healthy individuals after adjusting for age (p = 0.0149). Moreover, BER in control patients, but not in cases, was also lower than for healthy individuals (p = 0.0013). Maybe more meaningful, pre-aHCT patient samples did not show the correlation between NHEJ and SSA that was observed for every other group of samples analyzed, including the very same individuals at a time point post-aHCT (Table 3). Moreover, there was also absolutely no relationship of the NHEJ repair capacity to the patient’s age in pre-aHCT patients (r = 0.004, p = 0.984 –Table 5). Overall, this indicates several abnormalities in the DRC of pre-aHCT patient samples, which raises the question of the ability of such samples to reliably represent an individual’s repair capacity. It has been suggested that DRC in lymphocytes of cancer patients might be greatly affected by the general inflammation associated with the disease [57–59] or possibly by the cancer treatment itself. Consistent with that hypothesis, we had observed that DSB repair measurements by host-cell reactivation assays, even in lymphocytes from healthy individuals, could be influenced by factors in the environment of the cells, such as the presence of reactive oxygen species [34]. The DRC in lymphoma patients might be therefore more predictive of their outcome if it could be measured prior to any treatment, rather than after a large number of chemotherapy cycles, as is the case for the patients in this cohort that were recruited immediately prior to aHCT.

Regardless of what external factor might be influencing the repair measurements, the most striking results obtained from measuring DRC in our patient samples was the dramatic and quasi systematic decrease in both form of DSB repair associated with the aHCT itself (p<0.0001 for NHEJ and p = 0.0002 for SSA) when comparing the lymphocytes of the same individuals before and after transplant (Fig 4). The mechanism and cause of that decrease remains to be determined, and one cannot infer from our results anything about the repair in cells of the myeloid lineage, which are the ones involved in secondary t-MDS/AML. However, all blood cells, including lymphocytes and myeloid cells, derive from the same stem cells whether before or after the transplantation, and it is possible that the lower repair in peripheral blood lymphocytes is simply a manifestation of changes in hematopoietic stem cells in those individuals, just like the DSB repair in peripheral blood lymphocytes of healthy individuals was somehow the manifestation of these individuals’ age. CD34^+^ cells mobilized for aHCT in patients that later developed t-MDS/AML have been shown to present alterations in their DSB repair pathway as measured by microarray analysis [60] and it is possible that the repair deficiency post-aHCT was already present prior to the transplant, although possibly only in a subset of cells. Chemotherapy treatments of the primary lymphoma occurred prior to the harvesting of the hematopoietic stem cells and are likely to have resulted in the accumulation of DNA damage and subsequent mutations. Another major known factor that plays a role in accumulation of DNA damage in hematopoietic stem cells is ageing [61,62]. Patients undergoing aHCT regenerate marrow function from a limited number of mobilized stem cells [63] and this replication stress to replenish the blood marrow is also likely to lead *de novo* mutations in those cells. As a result, the decrease in DSB repair after aHCT might reflect the necessity to replicate extensively to reconstitute the whole hematopoietic system from a limited number of stem cells, or it could be that the transplant itself could form a selection mechanism for cells with lower repair capacity, whether directly or indirectly, through the ability to mobilize stem cells and/or to recolonize the bone marrow after aHCT. Investigating DRC in stem cell donors and recipients post-HCT in the context of allogeneic stem cell transplantations might be able to isolate specifically the effect of the transplant itself on DSB repair from other potential changes associated with chemotherapeutic treatments that might be relevant to autologous transplant cases. Either way, the lower repair post-transplant seemed to be a stable feature among the samples investigated (taken 100 days to 5 years post-aHCT–S1 Fig) as there was no trend for repair to decrease or increase within this time frame (data not shown). For those individuals with data both before and after aHCT, the average decrease in NHEJ of 6.9% associated with the transplant is higher than expected if caused by simple ageing. Based on the calculated rate of decrease of NHEJ repair observed for healthy individuals (0.37% decrease/year) in Fig 5, the average time difference of 21.6 months between the two measurements would be expected to lead to a decrease in NHEJ of only 0.65%, which is much lower than what was measured. One possibility is that transplanted stem cells show signs of accelerated ageing compared to never transplanted cells.

## Conclusions

We could not identify any difference in DRC existing prior to aHCT that could help predict which individuals are at higher risk of later developing t-MDS/AML. However, whether caused by the transplant itself and/or influenced by exposure to treatments, there is evidence that lymphocytes of patients post-transplant are greatly compromised in their ability to repair DSB. It remains to be determined whether the measured effect of transplantation on repair capacity is involved in making transplant patients, as a group, more susceptible for t-MDS/AML.

## Supporting information

S1 File Supporting Data DNA Repair Capacities. DNA repair capacities for each determination for different individuals using the assays described in the text.
(XLSX)

S1 Fig Samples used in the study. Each individual is represented by a code of 1 or 2 letter(s) (aHCT patients) or a combination H+number (healthy individuals). Commas separate each individuals’ code name. The nature of the sample (category of individual and/or time point) used is indicated. All samples indicated have some data represented in the study but we did not obtain data for all of those samples and/or for all of the tests performed. In all cases, samples analyzed were taken prior to any t-MDS/AML diagnosis. For more details on the data used, including pairing of specific t-MDS/AML cases to specific controls, refer to the file presenting the raw data (S1 File).
(TIF)

S2 Fig Quality control for DNA damage frequency in BER and NER plasmids templates for the assays. Host cell reactivation assay plasmid pM1-Luc was treated with methylene blue + visible light (MB) or UVC (UV) to generate damage classically repaired by BER (8-oxoG) or NER (pyrimidine dimers), respectively. The damage frequency generated by the treatment in the transcribed strand of firefly luciferase is quantified using 5 cycles of primer extension from a Cy5.5-labeled CMV-F primer (CGCAAATGGGCGGTAGGCGTG) using the LongAmp polymerase (New England Biolabs) on a BamHI-digested template. (**A**) Map of luciferase gene in pM1-Luc plasmid. (**B**) Cy5.5 signal after primer extension and 3.5% urea denaturating PAGE. The level of full length extension remaining on damaged templates (2.7kb) measures the proportion of plasmids undamaged in the luciferase coding sequence and can serve as quality control of damage level for each batch of plasmid generated for repair assays. The proportion of plasmid with blocking damage in the luciferase coding sequence can be inferred from the missing extensions as compared to the undamaged template control.
(TIF)

S3 Fig Comparison of DSB repair in unpurified lymphocytes (PBMCs) *vs* purified T cells. (**A**) NHEJ or (**B**) SSA repair in lymphocytes analyzed unpurified (PBMCs in black) or after purification of the CD3^+^ cell subpopulation (T cells in gray) for 5 separate healthy individuals.
(TIF)

S4 Fig Work flow for determination of repair capacity for all 4 pathways from a single aHCT patient cryopreserved sample. (TIF)

S5 Fig BER and NER before and after aHCT. (**A**) BER and NER measure in the same 18 individuals (9 controls, 9 cases) before and after aHCT (**B**) Repair post-aHCT normalized to pre a-HCT values for each individual. Mean value is indicated.
(TIF)

S6 Fig NER (red rectangle) and BER (black circle) repair capacity as a function of age in healthy individuals. 95% confidence intervals and trend lines are indicated.
(TIF)
