# Supplementary figures and images for: Autologous hematopoietic stem cell transplantation in lymphoma patients is associated with a decrease in the double strand break repair capacity of peripheral blood lymphocytes
Source: PLoS One. 2017 Feb 16;12(2):e0171473. doi: 10.1371/journal.pone.0171473 (PMC5313139; doi:10.1371/journal.pone.0171473)

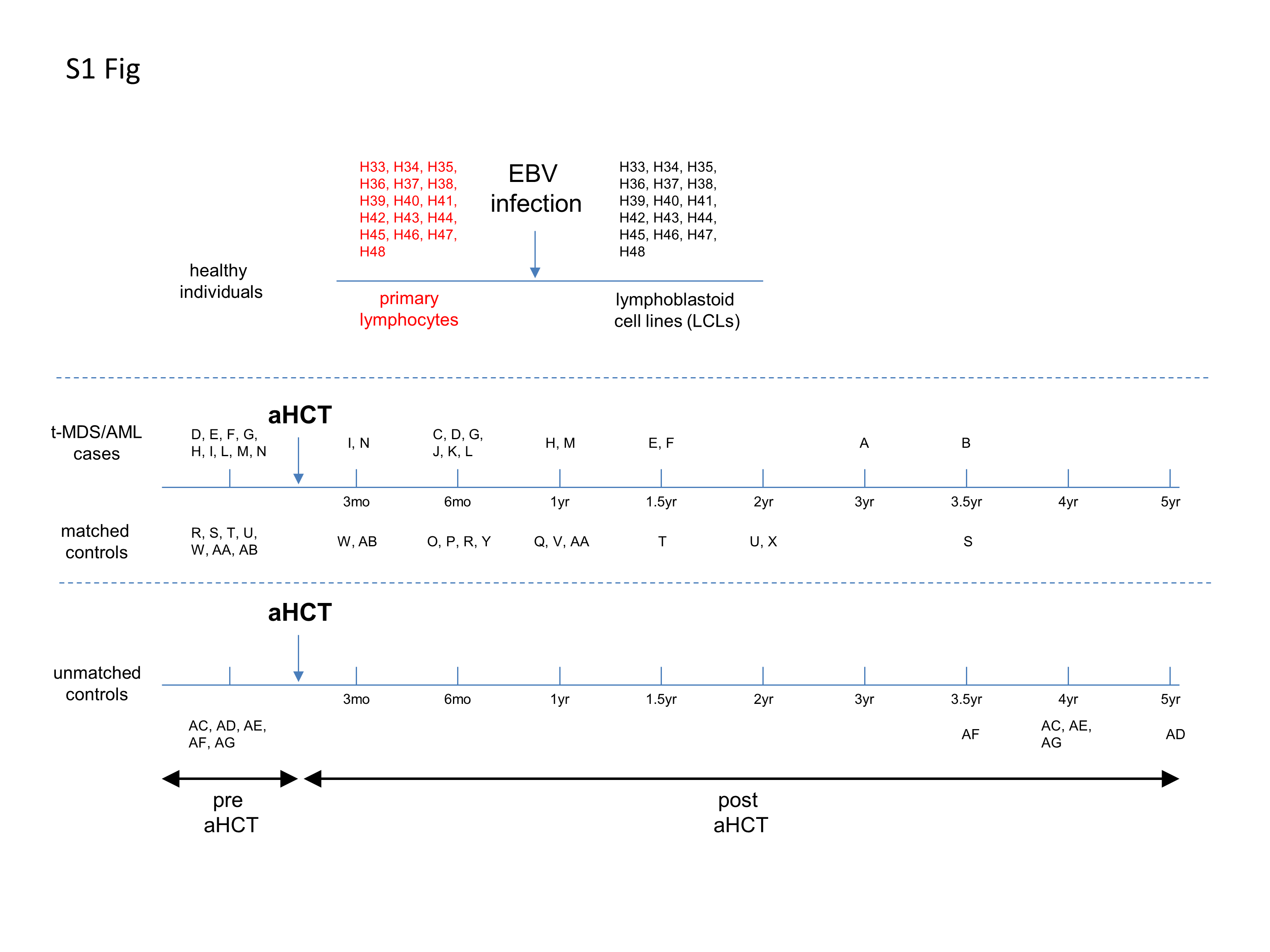

Supplement: S1 Fig — Each individual is represented by a code of 1 or 2 letter(s) (aHCT patients) or a combination H+number (healthy individuals). Commas separate each individuals’ code name. The nature of the sample (category of individual and/or time point) used is indicated. All samples indicated have some data represented in the study but we did not obtain data for all of those samples and/or for all of the tests performed. In all cases, samples analyzed were taken prior to any t-MDS/AML diagnosis. For more details on the data used, including pairing of specific t-MDS/AML cases to specific controls, refer to the file presenting the raw data (S1 File). (TIF) [file pone.0171473.s002.tif]

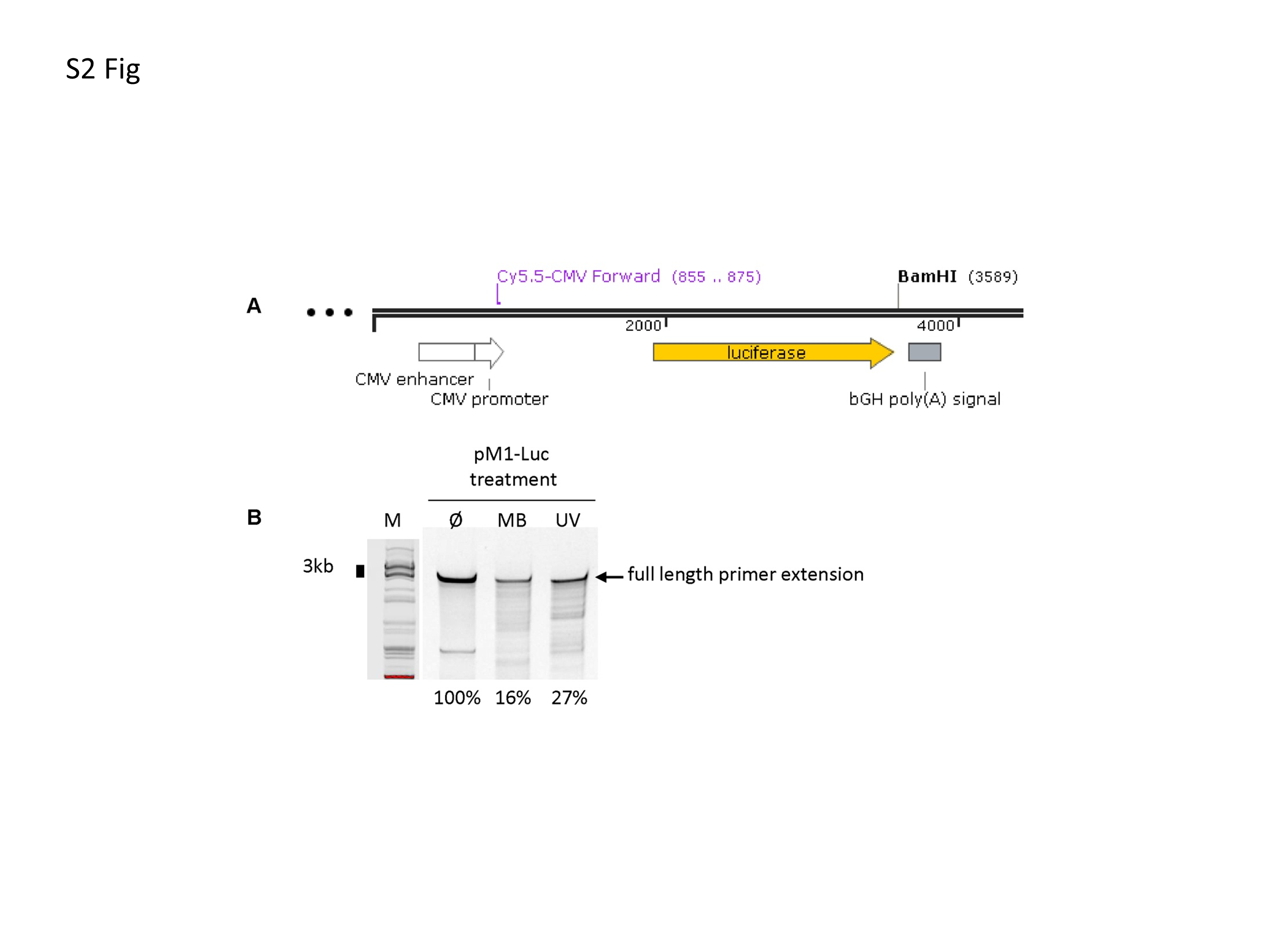

Supplement: S2 Fig — Host cell reactivation assay plasmid pM1-Luc was treated with methylene blue + visible light (MB) or UVC (UV) to generate damage classically repaired by BER (8-oxoG) or NER (pyrimidine dimers), respectively. The damage frequency generated by the treatment in the transcribed strand of firefly luciferase is quantified using 5 cycles of primer extension from a Cy5.5-labeled CMV-F primer (CGCAAATGGGCGGTAGGCGTG) using the LongAmp polymerase (New England Biolabs) on a BamHI-digested template. (A) Map of luciferase gene in pM1-Luc plasmid. (B) Cy5.5 signal after primer extension and 3.5% urea denaturating PAGE. The level of full length extension remaining on damaged templates (2.7kb) measures the proportion of plasmids undamaged in the luciferase coding sequence and can serve as quality control of damage level for each batch of plasmid generated for repair assays. The proportion of plasmid with blocking damage in the luciferase coding sequence can be inferred from the missing extensions as compared to the undamaged template control. (TIF) [file pone.0171473.s003.tif]

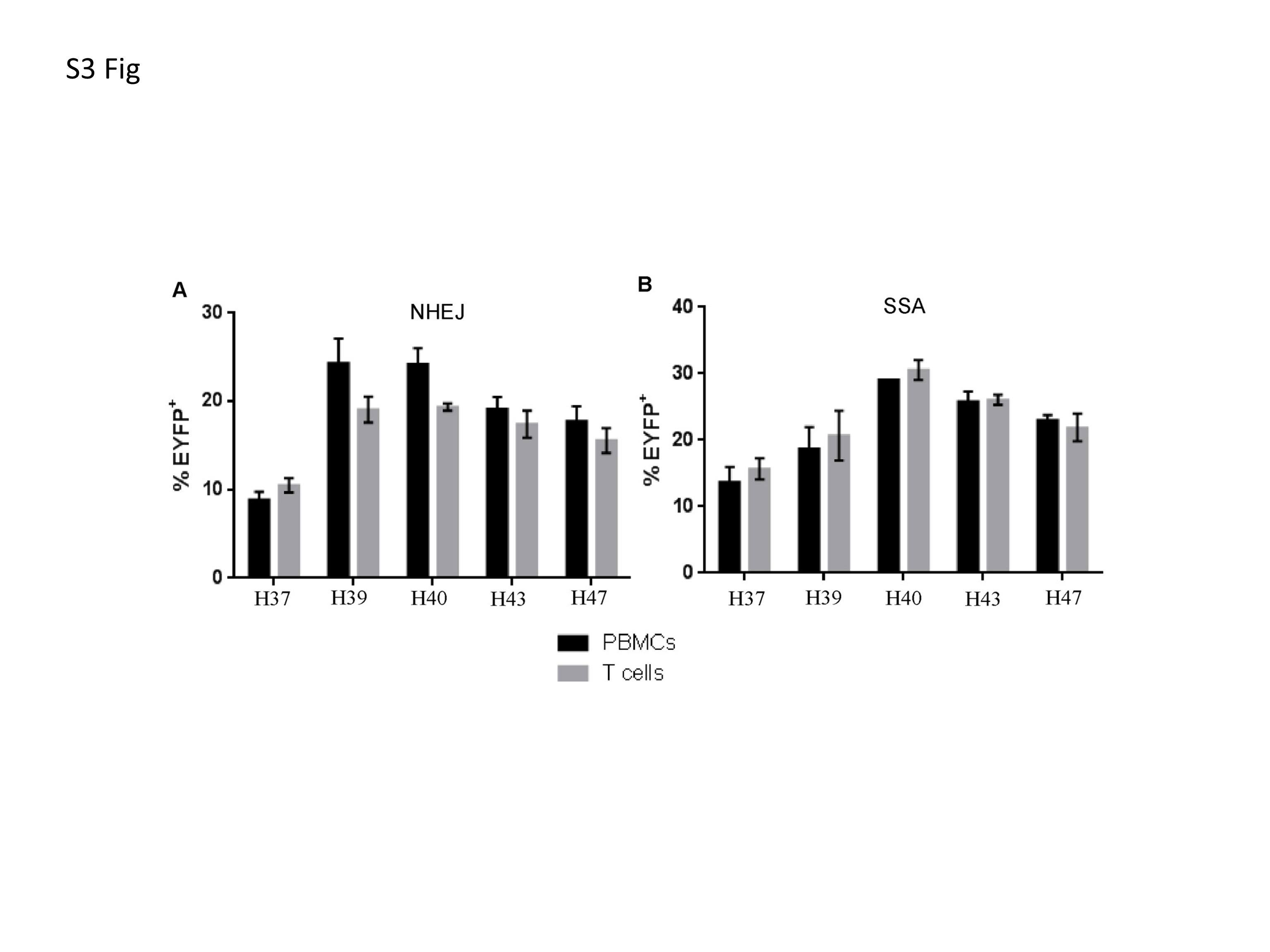

Supplement: S3 Fig — (A) NHEJ or (B) SSA repair in lymphocytes analyzed unpurified (PBMCs in black) or after purification of the CD3+ cell subpopulation (T cells in gray) for 5 separate healthy individuals. (TIF) [file pone.0171473.s004.tif]

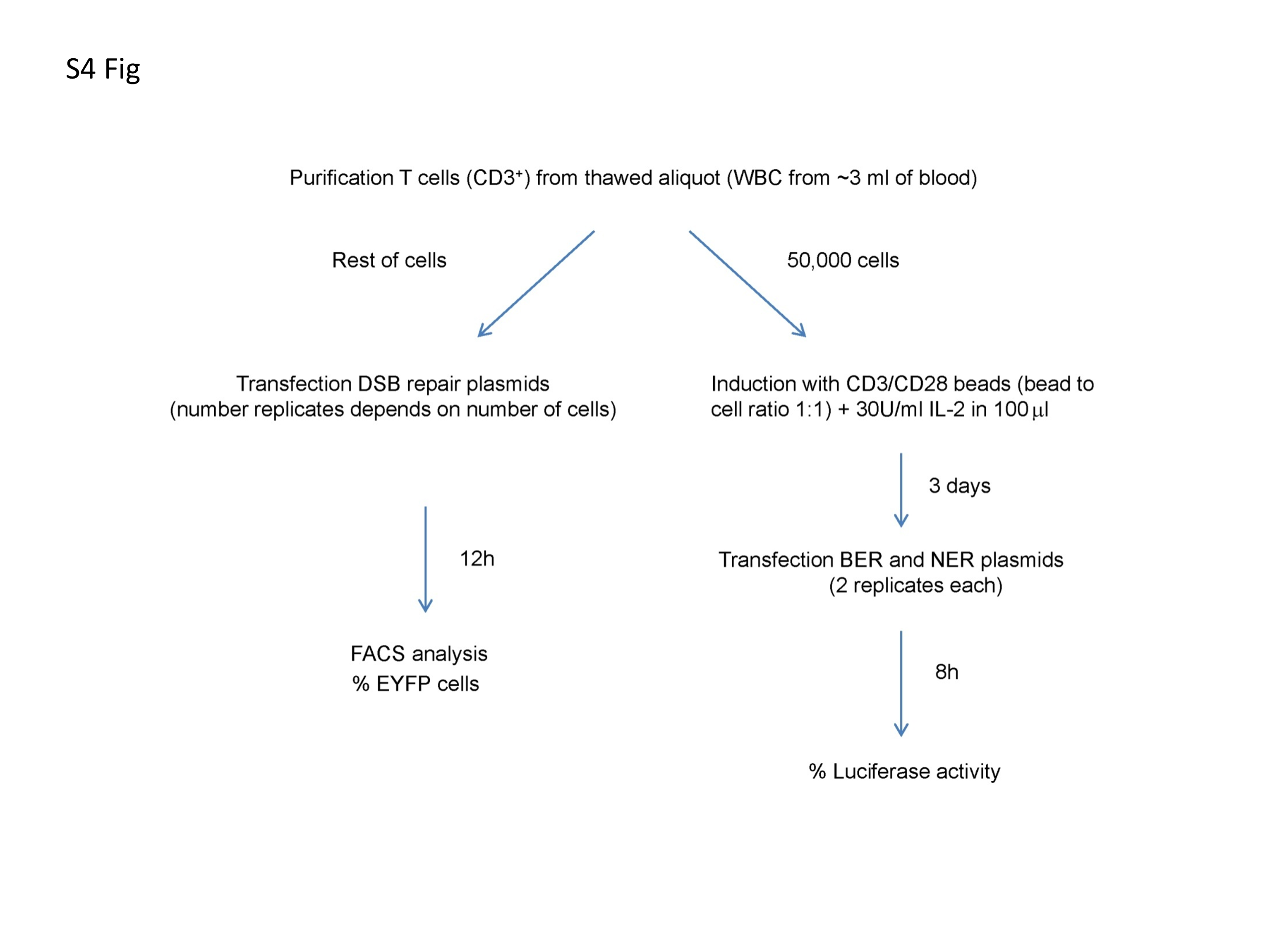

Supplement: S4 Fig — (TIF) [file pone.0171473.s005.tif]

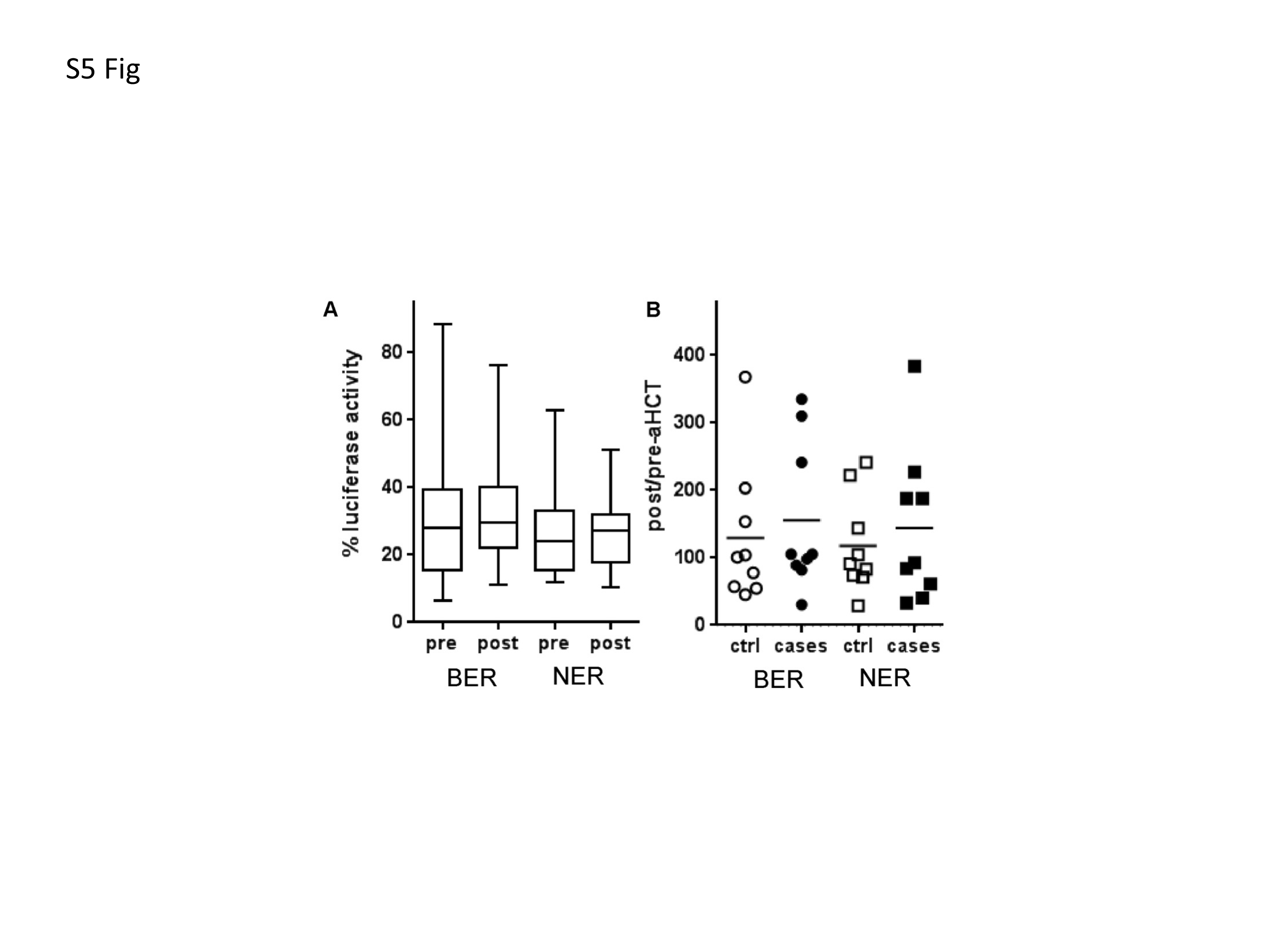

Supplement: S5 Fig — (A) BER and NER measure in the same 18 individuals (9 controls, 9 cases) before and after aHCT (B) Repair post-aHCT normalized to pre a-HCT values for each individual. Mean value is indicated. (TIF) [file pone.0171473.s006.tif]

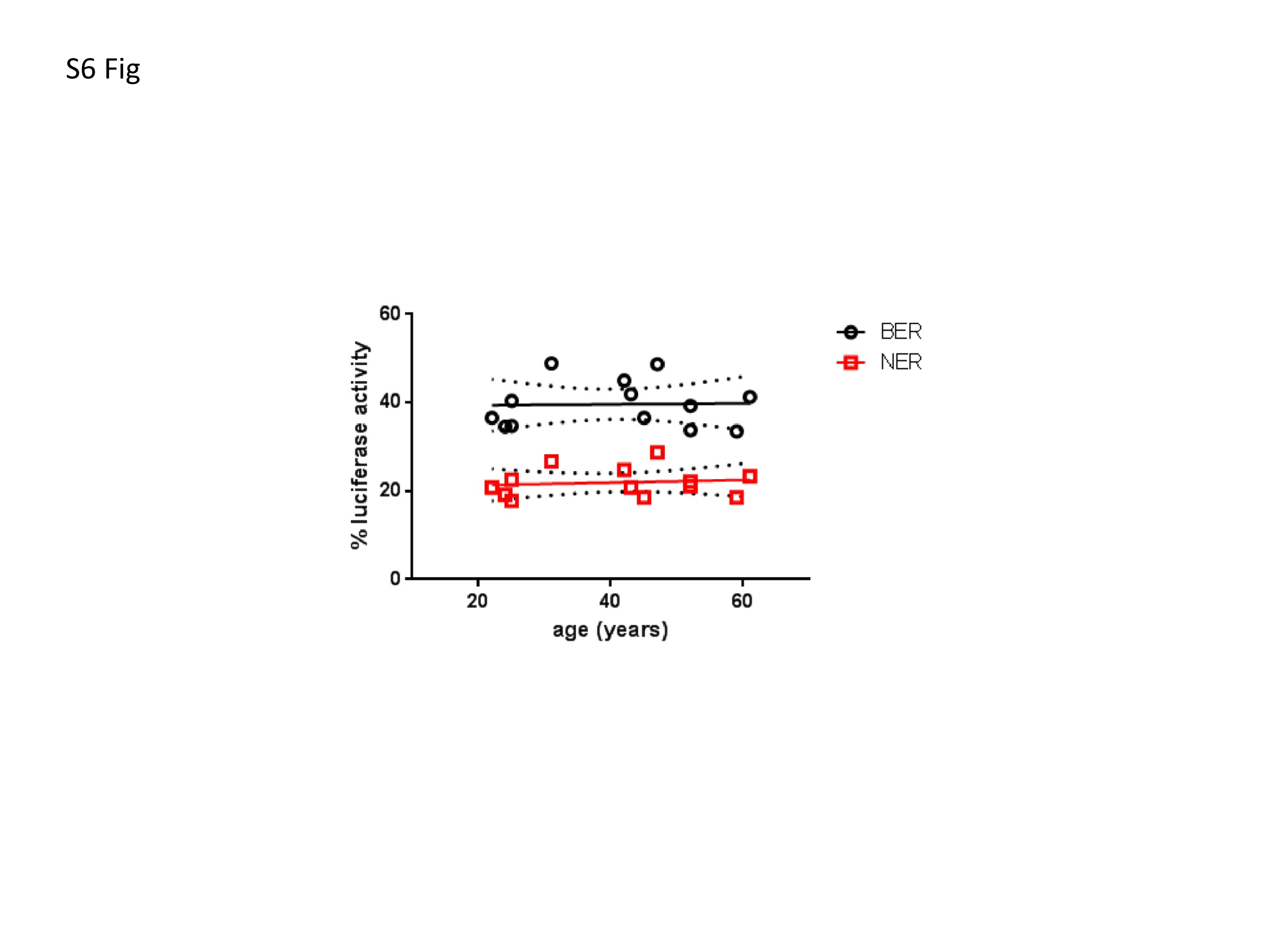

Supplement: S6 Fig — 95% confidence intervals and trend lines are indicated. (TIF) [file pone.0171473.s007.tif]
